# *MdTyDc* Overexpression Improves Alkalinity Tolerance in *Malus domestica*

**DOI:** 10.3389/fpls.2021.625890

**Published:** 2021-02-16

**Authors:** Xiaomin Liu, Yibo Jin, Kexin Tan, Jiangzhu Zheng, Tengteng Gao, Zhijun Zhang, Yongjuan Zhao, Fengwang Ma, Chao Li

**Affiliations:** State Key Laboratory of Crop Stress Biology for Arid Areas/Shaanxi Key Laboratory of Apple, College of Horticulture, Northwest A&F University, Xianyang, China

**Keywords:** *MdTyDc*, alkaline stress, dopamine, ion homeostasis, N metabolism

## Abstract

Tyrosine is decarboxylated to tyramine by TYDC (Tyrosine decarboxylase) and then hydroxylated to dopamine, which is involved in plant response to abiotic stress. However, little is known about the function of *MdTyDc* in response to alkaline stress in plants. In our study, it was found that the expression of *MdTyDc* was induced by alkaline stress. Therefore, the apple plants overexpressing *MdTyDc* was treated with alkali stress, and we found that *MdTyDc* played an important role in apple plants’ resistance to alkali stress. Our results showed that the restriction on the growth, the decrease of membrane permeability and the accumulation of Na^+^ were alleviated to various degrees in *MdTyDc* transgenic plants under alkali stress. In addition, overexpression of *MdTyDc* enhanced the root activity and photosynthetic capacity, and improved the enzyme activity related to N metabolism, thus promoting N absorption. It is noteworthy that the dopamine content of these three transgenic lines is significantly higher than that of WT. In summary, these findings indicated that *MdTyDc* may enhance alkaline tolerance of apples by mediating dopamine content, mainly by maintaining high photosynthetic capacity, normal ion homeostasis and strong nitrogen absorption capacity.

## Introduction

Soil salinization is one of the key abiotic stresses that inhibit plant growth; it significantly restricts agricultural production, especially in arid and semi-arid areas (Rozema and Flowers, 2008; Zhang et al., 2010). Alkaline stress (the stress caused by basic salts, primarily Na_2_CO_3_, and NaHCO_3_) includes components of osmotic stress, ion toxicity, and high pH stress; it greatly influences root function, photosynthesis, biological membranes, ion homeostasis, and nutrient metabolism, thereby strongly inhibiting plant growth (Yang et al., 2009; Guo et al., 2010, 2015a; Wang et al., 2015a; Sun et al., 2016; Liu et al., 2019a). Therefore, it is very important to improve the capacity of plants to resist alkaline stress.

Photosynthesis is an important physiological and biochemical process that supports plant growth and development. However, the photosynthetic apparatus are vulnerable to alkaline stress, leading to severe inhibition of photosynthesis (Xu et al., 2013; Li et al., 2020b). Alkaline stress hinders photosynthetic electron transfer, causing a decrease in apparent quantum yield and light energy conversion efficiency, thereby inhibiting PSII activity (Gorbe and Calatayud, 2012). In addition, alkaline stress can cause chlorophyll degradation, reduce chlorophyll content in thylakoid membranes, and inhibit the function of pigment–protein complexes (Yang et al., 2011).

The maintenance of intracellular ion balance and pH stability are necessary to ensure normal metabolism and energy conversion in plants. Alkaline stress significantly increases the content of sodium ions in the root zone, thereby causing serious osmotic stress to plants (Guo et al., 2010). Plants can respond to multiple ion stresses such as high Na^+^, low K^+^, high Mg^2+^, and high pH through specific salt overly sensitive (SOS) signal transduction pathways (Zhu, 2016). The known SOS system has four components: SOS1, SOS2, SOS3, and NHX (Zhu, 2016). The *SOS1* gene encodes a Na^+^/H^+^ antiporter (NHX) in the cell membrane that is responsible for sensing and expelling Na^+^ from the root (ElMahi et al., 2019). The SOS2/SOS3 complex negatively regulates the activity of NHX and high affinity K^+^ channels (HKT), positively regulates the activity of low affinity K channels (AKT), and promotes the absorption of K^+^ by root cells (Zhu, 2003). Under alkaline stress, plants can also absorb anions (primarily Cl^–^, SO_4_^2–^, NO_3_^–^, and organic acids) to balance the excess accumulation of cations in stems and leaves and maintain a stable pH (Yang et al., 2008). In addition, plants can alleviate osmotic stress by increasing their intracellular proline content and soluble sugar content (Flowers and Colmer, 2008; Dai et al., 2018).

Nitrogen (N) is one of the main elements that limit plant growth and crop yield, but plant N absorption can be significantly inhibited by low osmotic stress (Botella et al., 1997). Higher plants acquire N from both organic and inorganic sources, and the latter include NO_3_^–^ and NH_4_^+^ (Britto and Kronzucker, 2002; Bloom, 2015). Nitrate reductase (NR), nitrite reductase (NiR), glutamine synthetase (GS), and glutamic acid synthase (GOGAT) are key enzymes in the process of plant N uptake, transport, and assimilation (Luo et al., 2013). Previous work has shown that the damage caused by alkaline stress perturbs plant ion balance, impairing the activity of NR in cells, and thus inhibiting N absorption (Fujiyama, 2005). In addition, studies on N absorption and assimilation have been performed at the molecular and cellular levels, including studies of NH_4_^+^ transport by the ammonium transporter (AMT) protein family and NO_3_^–^ transport by the nitrate transporter (NRT) protein family (Wang et al., 2018). Moreover, the expression of NRT and AMT genes was shown to change in response to alkaline stress in rice (Wang et al., 2012a). In Arabidopsis NRT1, *AtNRT1.1*, and *AtNRT1.2* are mainly responsible for NO_3_^–^ uptake in roots (Orsel et al., 2007). *OsNRT1.1* and *OsNRT1.2* were up-regulated in old leaves under alkaline stress, suggesting that these two genes play an important role in NO_3_^–^ accumulation. *AtNRT2.4* is expressed in leaves and roots and is mainly involved in NO_3_^–^ transport from phloem to leaves (Kiba et al., 2012). *AtNRT2.5* is mainly located in the root epidermis and cortex, and its transcription is strongly induced by N starvation (Lezhneva et al., 2015). *AtNRT2.7* is located in the vacuolar membrane and its function is mainly to mediate NO_3_^–^ transport in the vacuole (Chopin et al., 2007). Huang et al. (2018) reported that members of the NRT family (*MdNRT1.1*, *MdNRT2.4*, *MdNRT2.5*, *MdNRT2.7*) were involved in the response to PEG-induced drought stress in apples. The roots of plants mainly absorb NH_4_^+^ through AMT. *AtAMT1.1*, *AtAMT1.2*, *AtAMT1.5*, *AtAMT2.1*, and *AtAMT3.1* are mainly expressed in root and aboveground parts (Sohlenkamp et al., 2000; Yuan et al., 2009). *AtAMT1.1* plays a leading role in the transport of NH_4_^+^ under N deficiency, and *AtAMT1.2* is mainly expressed in the endodermis of roots and is responsible for transporting NH_4_^+^ from roots to aboveground parts (Gazzarrini et al., 1999; Yuan et al., 2007). *PtrAMT1.5* and *PtrAMT1.6* play a certain role in plant reproductive development in *Populus trichocarpa* (Couturier et al., 2007). *AtAMT2.1* and *AtAMT2.2* has the function of high affinity ammonium transport in Arabidopsis (Sohlenkamp et al., 2002). The expression levels of *MdAMT3.1* and *MdAMT4.2* and *MdAMT4.3* in apple roots increased significantly with the decrease of N supply level (Huang et al., 2018).

Dopamine is a type of catecholamine that plays a vital role in plants, and various studies have shown that it has an important relationship with plant stress resistance. Li et al. (2015) demonstrated that dopamine relieved salt stress not only at the level of antioxidant defense but also through mechanisms such as the maintenance of ion balance. Exogenous dopamine improved the salt tolerance of apple by promoting its symbiosis with arbuscular mycorrhizal fungi (Gao et al., 2020a). In addition, dopamine has been shown to alleviate drought stress in apple seedlings through a variety of physiological mechanisms (Gao et al., 2020b). Liu et al. (2020) found that the absorption of mineral nutrients in apple seedlings was upregulated by exogenous dopamine, thereby promoting resistance to low N stress. Tyrosine decarboxylase (TYDC) is a key enzyme in the plant dopamine synthesis pathway (Nagatsu et al., 1972; Kong et al., 1998) and is involved in many plant secondary metabolism and defense processes. For example, *RcTyDc* can promote salidroside biosynthesis (Lan et al., 2013). Overexpression of *TYDC* in potato promoted the synthesis of tyrosine and effectively improved disease resistance (Landtag et al., 2002). Lehmann and Pollmann (2009) showed that the expression of *TYDC* in *Arabidopsis* was induced by drought stress and injury.

Previously, we found that dopamine content was increased by the overexpression of *MdTyDc* in apple seedlings and calli, and apple salt tolerance was improved by its overexpression in apple plants (Wang et al., 2020). Moreover, previous studies in our lab showed that the expression of N-metabolism-related genes in apple seedlings was up-regulated by exogenous dopamine under alkaline stress, permitting better adaptation to this stress (Jiao et al., 2019). Although the effects of TYDC on plant growth and resistance to biotic and abiotic stress have been described, the mechanism by which TYDC influences plant response to alkaline stress is not fully understood. Here, we found that overexpression of *MdTyDc* improved the ability of apples to cope with alkaline stress. This study adds to our understanding of the mechanisms by which *MdTyDc* functions in response to alkaline stress.

## Materials and Methods

### Plant Materials and Alkaline Treatments

Our experiments were carried out at the Northwest A&F University, Yangling (34°20′ N, 108°24′ E) in Shaanxi, China. WT and transgenic GL-3 (*Malus domestica* cv. Gala) plants were obtained from the previous research in our laboratory (Wang et al., 2020). After subculturing and rooting, the apple plants were transplanted to plastic pots (12 × 12 cm) filled with soil/perlite/vermiculite (4:1:1, v:v:v) and placed in a growth chamber. WT and transgenic apple plants at the same growth stage (7–9 true leaves) were transferred to plastic containers (52 × 37 × 15 cm^3^) that were wrapped in black plastic and contained 13 L half-strength Hoagland’s nutrient solution. The pH of the nutrient solution was adjusted to 6.0 ± 0.2 with H_3_PO_4_ or KOH, and it was changed every 5 days. Our hydroponic culture system was designed and used as described in Li et al. (2012). The apple plants were cultivated in a growthchamber where the growth conditions included a 14 h photoperiod (the light intensity was 160 μmol m^–2^ s^–1^), 24 ± 2°C/16 ± 2°C day/night and 60 ± 5% relative humidity. After 12 days of pre-cultivation, WT and transgenic GL-3 plants of uniform size were selected and randomly divided into two groups: (1) the control group (CK) that received half-strength Hoagland’s nutrient solution with a pH of 6.0 ± 0.2 and (2) the alkaline stress group (AL) that received half-strength Hoagland’s nutrient solution with a pH of 9.0 ± 0.2. The solution pH was adjusted with 1 *M* NaHCO_3_ and 1 *M* Na_2_CO_3_ (1:1), and the treatment lasted for 15 days.

### Measurement of Dopamine Contents

A 0.1 g sample of fresh leaf and root tissues was weighed and placed in a 2 mL centrifuge tube, and 1 mL acidic methanol (hydrochloric acid:methanol = 1:9, v/v) was added. After mixing well, all samples were centrifuged at 3,500 rpm for 5 min. The supernatant was filtered through a 0.45 μm membrane and analyzed by high-performance liquid chromatography (HPLC, LC-2010, Shimadzu, Japan).

### Growth Measurements

At the beginning and end of the alkaline stress treatment, the growth indices of all plants were measured. The stem height (SH) was measured with a plastic ruler, and the leaf number (LN) was counted. All the harvested plants were divided into three parts: roots, stems, and leaves. Total fresh weight (TFW), total dry weight (TDW), root–shoot ratio (RSR), and relative growth rate (RGR) were calculated as described previously (Liang et al., 2017).

### Root Architecture Measurements

Root systems were carefully cleaned without damaging the roots. After the roots had been flattened and spread out, a SNAPSCAN 310 scanner was used to obtain their images. Finally, root system parameters were analyzed with WinRHIZO image analysis software (V4.1c; Regent Instruments, Quebec City, QC, Canada).

### Measurement of Root Activity and Relative Electrolyte Leakage

Root vital staining was performed using the triphenyl tetrazolium chloride (TTC) method of Joseph et al. (2010), and the dehydrogenase activity (mg g^–1^ FW h^–1^) was used to represent root activity. Relative electrolyte leakage (REL) was measured by the method of Dionisio-Sese and Tobita (1998).

### Quantification of Photosynthetic Characteristics

The net photosynthesis rate (Pn), intercellular CO_2_ concentration (Ci), stomatal conductance (Gs), and transpiration rate (Tr) were recorded from 9:00 to 11:00 a.m. with a CIRAS-3 portable photosynthesis system (CIRAS-3, PP Systems, Amesbury, United States). All photosynthetic characteristics were measured at 1,000 μmol photons m^–2^ s^–1^ and a constant 500 μmol s^–1^ airflow rate. The CO_2_ concentration was 400 μmol CO_2_ mol^–1^ air. For each treatment, photosynthetic parameters were measured from 10 fully exposed and mature leaves at the same position.

### Chlorophyll Content and Fv/Fm Measurements

According to the method of Arnon (1949), the cleaned leaves were cut into filaments of about 0.1 cm with scissors. 0.1 g of the mixed filaments were weighed and loaded into a 10 mL centrifuge tube containing 8 mL 80% acetone. After shaking evenly, they were placed in a dark place (room temperature, 24 h) and shaken 3–4 times during the period. The optical density values at the wavelengths of 663, 645, and 470 nm were determined by UV-2250 spectrophotometer (Shimadzu, Kyoto, Japan).

For chlorophyll fluorescence measurements, black cloth was used to cover fresh, mature leaves at the same position on plants from each treatment for 30 min. Fv/Fm (the maximum quantum efficiency of photosystem II) was measured using a three-dimensional chlorophyll fluorescence imaging system (FC800, PSI, Czech Republic). The parameters of this system were set as follows: the shutter value was 1; the light source was flashes; and the sensitivity value was 70%.

### Measurements of Inorganic Ion and Proline Content

The apple plants were washed with tap water, distilled water and double distilled water successively, and the water on the surface of plants was carefully dried with filter paper. Then the plants were divided into root, stem and leaf parts. After 30 min of high temperature treatment at 105°C, the plants were placed at 80°C for at least 72 h to constant weight. After drying, the samples were broken and bagged with a pulverizer for the determination of mineral element content. 0.1 g of dry sample was weighed and placed into a centrifuge tube with 20 mL of deionized water. Then it was extracted at 100°C for 20 min for the determination of ions contents. The contents of Na^+^ and K^+^ were determined by flame photometry (FP6410 flame photometer). The contents of nitrate, chloride, sulfate, dihydrogen phosphate, and oxalic acid Cl^–^, SO_4_^2–^, and NO_3_^–^ were determined by ion chromatography (Thermo Fisher Scientific DIONEX ICS-1100). 0.1 g of dry sample was weighed and placed into a desiccating tube containing 5 mL concentrated sulfuric acid (H_2_SO_4_, AR, 98%). H_2_O_2_ was added for deboiling according to the digestion method of plant sample. After disboiling, the digestion solution was filled with deionized water at constant volume to 100 mL, and the supernatant was to be measured. The content of N was determined by AA3 continuous flow analyzer.

Proline (Pro) was extracted and measured according to the method of Jin et al. (2019), and Pro content was analyzed using liquid chromatography–mass spectrometry (LC–MS, LC: AC, ExionLC; MS:Q-trap5500, AB Sciex Pret., Ltd., Framingham, MA, United States). Methanol and 0.1% methanolic acid were used as mobile phases A and B, respectively. The flow rate was set to 0.3 mL min^–1^. The injection volume is 10 μl. The retention time of Pro was 12 min, and the content of Pro was calculated using the peak area of the standard curve.

### Measurement of Enzyme Activities

Three apple plants were randomly sampled from each treatment on the 15th day after alkaline stress treatment. They were washed with distilled water and dried with filter paper. After the leaves and roots of each plant were separated, they were mixed separately and immediately put into liquid nitrogen. Then they were placed in the −80°C refrigerator for testing. The activity of nitrate reductase (NR) was determined by the method of Högberg et al. (1986), the activity of nitrite reductase (NiR) was measured according the previous study (Seith et al., 1994), the activity of glutamine synthase (GS) was analyzed by spectrophotometry (Wang et al., 2008) and the activity of glutamate synthase (GOGAT) by was tested based on the method of Lin and Kao (1996).

### qRT–PCR Analysis

Total RNA was extracted from leaf and root samples using the Wolact plant RNA extraction kit (Vicband, Hong Kong, China). Complementary DNA was then reverse transcribed using the PrimeScript RT reagent Kit with gDNA Eraser (Perfect Real Time). The real-time quantitative PCR (qRT-PCR) reactions were carried out using the SYBR Green qPCR kit (TaKaRa, Tokyo, Japan) and the iQ5 Multicolor Detection System (Bio-Rad Laboratories, Hercules, United States). Perini et al. (2014) showed that *MdMDH* (malate dehydrogenase gene) were found to be the most stable and suitable normalizers for all apple tissue expression analyses by RT-qPCR. Therefore, MDH was used as an internal standard. The specific primer sequences used for qRT–PCR are provided in Supplementary Table S1. At 0, 3, 12, 24 h, 3, 5, 10, and 15 days after alkali stress treatment, leaves and roots were randomly selected for measurement.

### Statistical Analysis

The results are presented as means ± SD. The differences between treatments were evaluated by Tukey’s test after a one-way ANOVA (*P* < 0.05) with SPSS 25.0 for Windows.

## Results

### Overexpression of *MdTyDc* Alleviates Alkaline Stress in Apple

**FIGURE 1:**
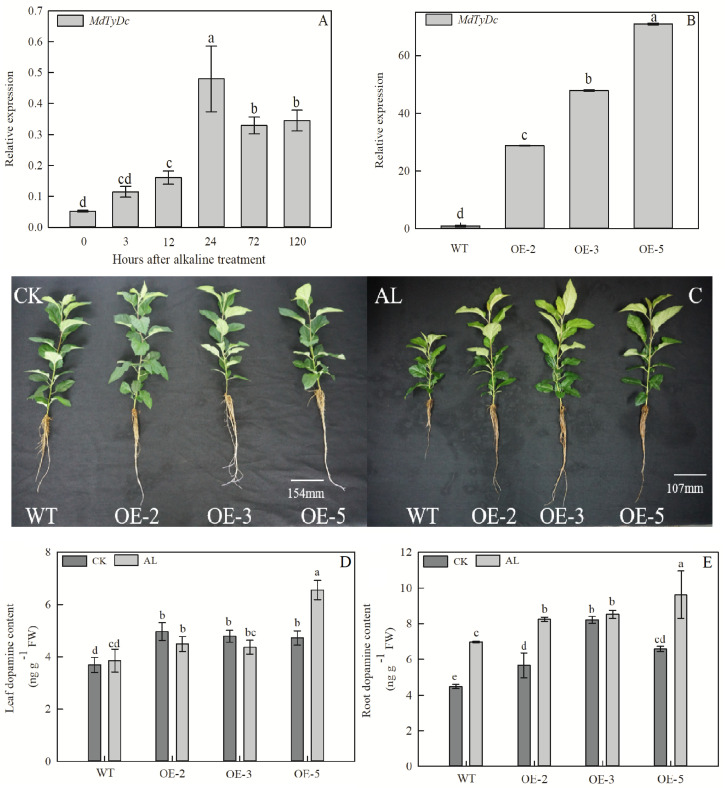
Overexpression of *MdTyDc* confers enhanced alkaline tolerance to apple. **(A)** The expression of *MdTyDc* after alkaline treatment in GL-3 apple; **(B)** qRT–PCR analysis of *MdTyDc* transcripts in lines WT, OE-2, OE-3, and OE-5; **(C)** the phenotype and dopamine content of *MdTyDc*-overexpressing apple plants in **(D)** leaves and **(E)** roots after 15 days under control and alkaline conditions. The data are presented as means ± SD (*n* = 3). Significant differences between WT and *MdTyDc* overexpression lines are indicated by different lowercase letters based on Tukey’s multi-range test (*P* < 0.05). WT, wild type. OE-2, *MdTyDc* overexpression line 2. OE-3, *MdTyDc* overexpression line 3. OE-5, *MdTyDc* overexpression line 5.

*MdTyDc* expression was induced by alkaline treatment of GL-3 apple plants (Figure 1A), and three overexpressing (OE) apple lines were used to investigate the role of *MdTyDc* under alkaline stress. In OE-2, OE-3, and OE-5 lines, *MdTyDc* transcripts were elevated 28. 81-, 47. 89-, and 70.87-fold compared with WT (Figure 1B).

The growth of apple plants was severely inhibited after 15 days of alkaline stress; however, OE lines were more tolerant to alkaline stress than WT (Figure 1C). Plant height and leaf number were significantly higher in OE lines than in WT under alkaline conditions (Table 1). The TFW, TDW, and RGR of all genotypes were not significantly different under normal hydroponics conditions. However, these parameters were notably lower in WT plants than OE lines under alkaline stress (Table 1). In addition, the WT plants showed clear increases in RSR compared with OE plants under stress treatment (Table 1). All these results indicated that *MdTyDc* plays an important role in the response to alkaline stress.

**TABLE 1:** Stem height (SH), leaf number (LN), total fresh weight (TFW), total dry weight (TDW), relative growth rate (RGR), and root stem ratio (RSR) of MdTyDc-overexpressing apple plants after 15 days under control and alkaline conditions.

Treatment	SH (cm)	LN (No. plant^–1^)	TFW (g plant^–1^)	TDW (g plant^–1^)	RGR (%)	RSR (%)
CK-WT	33.14 + 2.78ab	21.40 + 1.26b	9.98 + 0.58a	1.91 + 0.19a	76.92 + 6.48a	14.83 + 2.23cd
CK-OE-2	34.57 + 3.47a	21.50 + 1.51b	9.70 + 0.8a	1.84 + 0.34ab	67.11 + 11.44ab	16.19 + 3.44bcd
CK-OE-3	31.86 + 4.56ab	23.60 + 2.12a	9.57 + 1.21a	1.98 + 0.47a	73.61 + 15.91a	14.9 + 3.68cd
CK-OE-5	30.82 + 3.26b	22.10 + 1.79b	9.34 + 0.47a	1.98 + 0.23a	71.81 + 8.63a	14.26 + 2.57d
AL-WT	13.53 + 1.77e	16.20 + 1.03d	2.65 + 0.56c	0.94 + 0.15d	28.86 + 10.37d	23.43 + 4.81a
AL-OE-2	22.48 + 4.02c	17.90 + 1.91c	4.97 + 1.53b	1.52 + 0.42c	50.42 + 16.62c	19.07 + 3.79b
AL-OE-3	19.72 + 2.08d	190 + 1.76c	5.03 + 0.73b	1.53 + 0.19c	57.79 + 8.18bc	17.59 + 1.50bc
AL-OE-5	19.15 + 1.15d	18.30 + 1.25c	4.83 + 0.48b	1.6 + 0.23bc	57.72 + 8.71bc	17.24 + 2.75bcd

*The data are presented as means ± SD (n = 10). Significant differences between WT and MdTyDc overexpression lines are indicated by different lowercase letters based on Tukey’s multi-range test (P < 0.05). WT, wild type. OE-2, MdTyDc overexpression line 2. OE-3, MdTyDc overexpression line 3. OE-5, MdTyDc overexpression line 5.*

### Overexpression of *MdTyDc* Increased Dopamine Content

Because tyrosine decarboxylase is a vital enzyme in the plant dopamine synthesis pathway, plant dopamine contents were measured. The content of dopamine in leaves and roots of OE lines was significantly higher than that of WT lines under both normal and alkaline stress conditions (Figures 1D,E and Supplementary Figure S1). Moreover, after 5 days of alkaline treatment, the dopamine content in the leaves and roots of WT and OE plants was markedly increased due to the damage caused by alkaline stress (Supplementary Figure S1). These data showed that the overexpression of *MdTyDc* increases dopamine content in apple plants under both normal and alkaline conditions.

### Overexpression of *MdTyDc* Affected Root Development, REL, and Proline Content Under Alkaline Conditions

After 15 days of alkaline treatment, the root architecture significantly changed, although WT and OE lines showed no significant differences under normal hydroponics conditions. The root length, surface area, number of root tips, and number of root forks were less affected by alkaline stress in transgenic apple plants than in WT plants (Table 2). Furthermore, under alkaline stress, the average root diameter of WT plants was noticeably smaller than that of OE-2 and OE-3 plants, and the root volume of OE-2 and OE-5 plants was significantly higher than that of WT plants (Table 2).

**TABLE 2:** Root system architecture of *MdTyDc*-overexpression apple plants after 15 days under control and alkaline conditions.

Treatment	Length (cm)	Avg diam (mm)	Volume (cm^3^)	Tips (No. root^–^^1^)	Forks (No. root^–^^1^)	Surf area (cm^2^)

	M ± SD	M ± SD	M ± SD	M ± SD	M ± SD	M ± SD
CK-WT	529.81 + 29.35ab	0.43 + 0.01ab	0.87 + 0.003a	1030.65 + 5.65b	3846.03 + 40.24a	85.33 + 1.28a
CK-OE-2	518.6 + 45.42b	0.43 + 0.01a	0.86 + 0.001ab	1039.19 + 5.29a	3887.88 + 26.98a	85.86 + 0.4a
CK-OE-3	561.92 + 18.78a	0.43 + 0.01ab	0.86 + 0.002a	1031.96 + 2.91b	3894.92 + 19.86a	86.29 + 0.62a
CK-OE-5	556.5 + 24.56a	0.43 + 0.01ab	0.86 + 0.005a	1033.33 + 3.59ab	3895.09 + 57.57a	85.7 + 1.45a
AL-WT	329.4 + 21.12d	0.43 + 0.01b	0.86 + 0.004b	930.23 + 5.4d	2591.78 + 36.06c	65.32 + 0.43d
AL-OE-2	454.23 + 17.11c	0.43 + 0.01a	0.86 + 0.005a	983.9 + 4.25c	3596.26 + 56.5b	77.03 + 1.17b
AL-OE-3	438.02 + 9.47c	0.43 + 0.01a	0.86 + 0.004ab	983.16 + 5.11c	3598.9 + 58.3b	75.2 + 1.84c
AL-OE-5	434.29 + 14.04c	0.43 + 0.01ab	0.86 + 0.005a	987.7 + 4.44c	3646.23 + 39.16b	76.08 + 0.86bc

*The data are presented as means ± SD (n = 10). Significant differences between WT and MdTyDc overexpression lines are indicated by different lowercase letters based on Tukey’s multi-range test (P < 0.05). WT, wild type. OE-2, MdTyDc overexpression line 2. OE-3, MdTyDc overexpression line 3. OE-5, MdTyDc overexpression line 5.*

**FIGURE 2:**
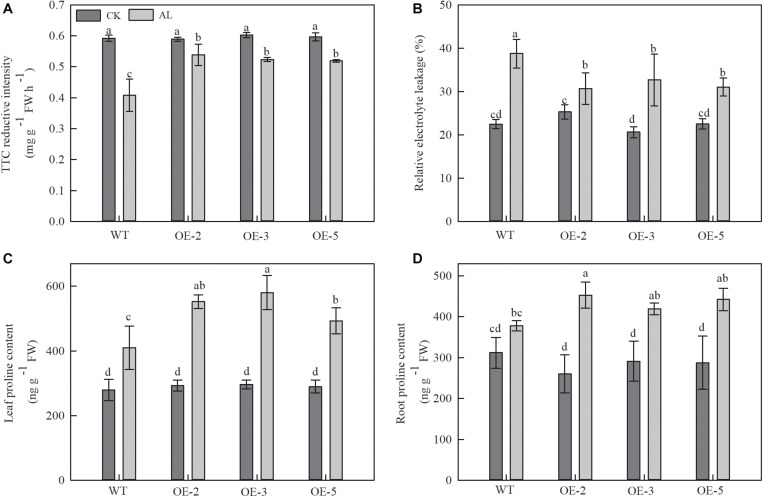
Overexpression of *MdTyDc* had an effect on the root development, REL and proline content after 15 days under control and alkaline conditions. **(A)** The root activity, **(B)** leaf relative electrolyte leakage (REL), **(C)** leaf proline content, and **(D)** root proline content. The data are presented as means ± SD (*n* = 3). Significant differences between WT and *MdTyDc* overexpression lines are indicated by different lowercase letters based on Tukey’s multi-range test (*P* < 0.05). WT, wild type. OE-2, *MdTyDc* overexpression line 2. OE-3, *MdTyDc* overexpression line 3. OE-5, *MdTyDc* overexpression line 5.

TTC reduction was used as an indicator of root activity. TTC reduction decreased in WT plants after 15 days of alkaline treatment, but this decrease was noticeably mitigated in OE plants. TTC reduction measurements from OE-2, OE-3, and OE-5 plants were 32.01, 28.27, and 27.16% higher than those of WT plants (Figure 2A).

Relative electrolyte leakage (REL) from leaves was used to assess the influence of alkaline stress on leaf membrane permeability. Under alkaline treatment, REL was 15.67–20.82% lower in the OE lines than in the WT (Figure 2B). These findings suggested that *MdTyDc* transgenic lines had stronger root systems and leaf membrane systems than WT lines under alkaline stress.

We also measured the content of proline, which is considered to be the main osmotic regulator. Proline content increased in all apple plants under alkaline stress (Figures 2C,D), but that of OE plants was noticeably higher than that of WT plants (Figure 2C). A similar trend was observed for root proline contents under alkaline stress. Although the differences did not reach the threshold for statistical significance, the root proline content of OE-2, OE-3, and OE-5 lines was 19.88, 11.03, and 17.07% higher than that of WT lines (Figure 2D). These results showed that the tolerance of *MdTyDc* transgenic lines to alkaline stress was related to balanced ion homeostasis and increased proline accumulation.

### Apple Lines That Overexpressed *MdTyDc* Maintained a Stronger Photosynthetic System Under Alkaline Stress

**FIGURE 3:**
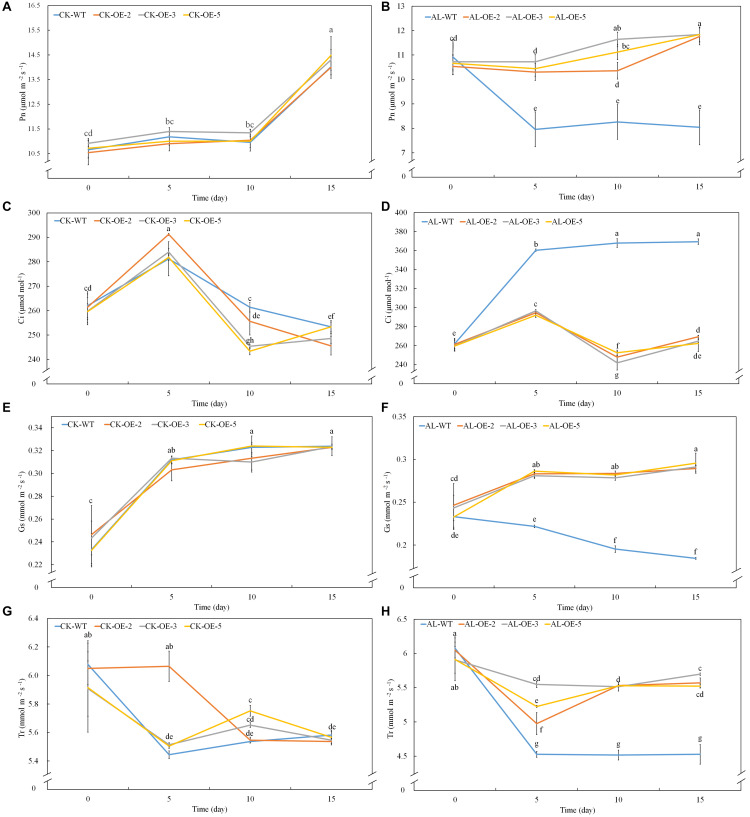
Apple lines overexpressing *MdTyDc* maintain a stronger photosynthetic system after 15 days under control and alkaline conditions. **(A,B)** Net photosynthesis (Pn) under **(A)** control and **(B)** alkaline conditions, **(C,D)** intercellular CO_2_ concentration (Ci) under **(C)** control and **(D)** alkaline conditions, **(E,F)** stomatal conductance (Gs) under **(E)** control and **(F)** alkaline conditions, and **(G,H)** transpiration rate (Tr) under **(G)** control and **(H)** alkaline conditions. The data are presented as means ± SD (*n* = 5). Significant differences between WT and *MdTyDc* overexpression lines are indicated by different lowercase letters based on Tukey’s multi-range test (*P* < 0.05). WT, wild type. OE-2, *MdTyDc* overexpression line 2. OE-3, *MdTyDc* overexpression line 3. OE-5, *MdTyDc* overexpression line 5.

**FIGURE 4:**
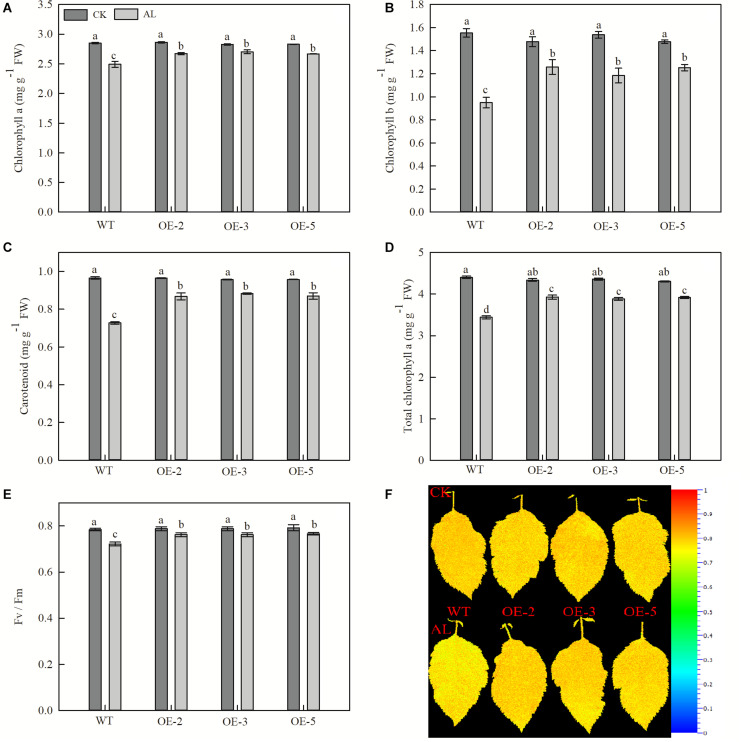
Overexpression of *MdTyDc* enhanced the chlorophyll concentrations and Fv/Fm after 15 days under control and alkaline conditions. **(A)** Chlorophyll a, **(B)** chlorophyll b, **(C)** carotenoids, **(D)** total chlorophyll, **(E)** Fv/Fm, and **(F)** Fv/Fm image obtained from chlorophyll fluorescence imaging. The data are presented as means ± SD (*n* = 5). Significant differences between WT and *MdTyDc* overexpression lines are indicated by different lowercase letters based on Tukey’s multi-range test (*P* < 0.05). WT, wild type. OE-2, *MdTyDc* overexpression line 2. OE-3, *MdTyDc* overexpression line 3. OE-5, *MdTyDc* overexpression line 5.

Net photosynthetic rate is another key index of plant growth. The WT and transgenic lines showed no conspicuous differences in Pn, Ci, Gs, or Tr under normal growing conditions (Figures 3A,C,E,G). Pn declined rapidly from the fifth day of alkaline treatment, but the overexpression of *MdTyDc* slowed this decline noticeably (Figure 3B). The Ci values were markedly higher for OE plants than for WT plants under alkaline conditions (Figure 3D), and Gs and Tr showed a trend similar to that of Pn (Figures 3F,H). Chlorophyll a, chlorophyll b, carotenoid, and total chlorophyll contents also decreased in response to alkaline stress, but this reduction was mitigated in the transgenic lines (Figures 4A–D).

The physiological activity of PSII influences chlorophyll fluorescence, and we therefore measured Fv/Fm, which represents the maximum photochemical efficiency of PSII photochemistry. After 15 days of alkaline stress, Fv/Fm decreased by 7.91% in WT plants and by ∼3.29% in the three OE lines (Figures 4E,F). Together, these data indicated that the overexpression of *MdTyDc* enhanced photosynthetic activity, thereby increasing plant resistance to alkaline stress.

### *MdTyDc* Overexpression Changed Inorganic Ion Content

**FIGURE 5:**
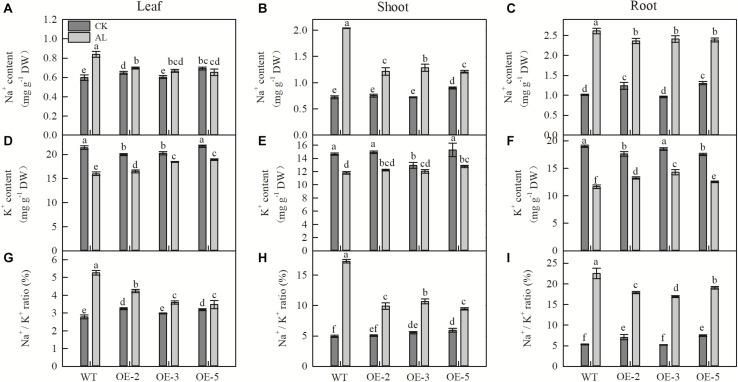
*MdTyDc* overexpression changed the Na^+^and K^+^ content in apple plants after 15 days under control and alkaline conditions. **(A)** Leaf Na^+^ content, **(B)** shoot Na^+^ content, **(C)** root Na^+^ content, **(D)** leaf K^+^ content, **(E)** shoot K^+^ content, **(F)** root K^+^ content, **(G)** leaf Na^+^/K^+^ ratio, **(H)** shoot Na^+^/K^+^ ratio, and **(I)** root Na^+^/K^+^ ratio. The data are presented as means ± SD (*n* = 3). Significant differences between WT and *MdTyDc* overexpression lines are indicated by different lowercase letters based on Tukey’s multi-range test (*P* < 0.05). WT, wild type. OE-2, *MdTyDc* overexpression line 2. OE-3, *MdTyDc* overexpression line 3. OE-5, *MdTyDc* overexpression line 5.

Na^+^ content of leaves, shoots, and roots was strongly increased after 15 days of alkaline treatment. However, the Na^+^ content of these tissues in apple *MdTyDc* OE lines was significantly lower than that of WT plants (Figures 5A–C). Leaf and root K^+^ content decreased after alkaline treatment, but this decrease was considerably smaller in the three OE lines than in WT plants, and there was no significant difference in stem K^+^ content between WT and OE lines (Figures 5D–F). Changes in the Na^+^/K^+^ ratio of leaves, stems, and roots followed a trend similar to that of Na^+^ content (Figures 5G–I).

**FIGURE 6:**
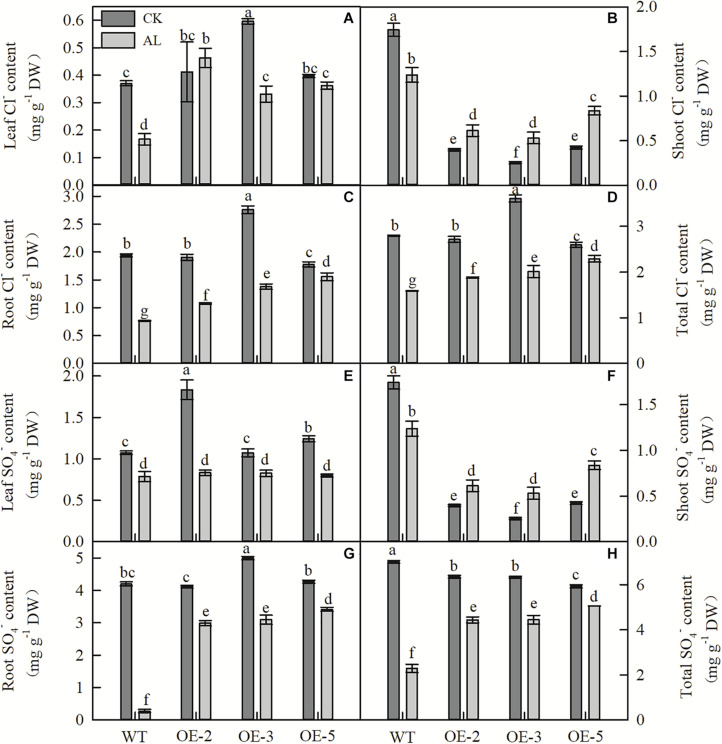
*MdTyDc* overexpression changed the Cl^–^and SO_4_^2–^ content in apple plants after 15 days under control and alkaline conditions. **(A)** Leaf Cl^–^ content, **(B)** shoot Cl^–^ content, **(C)** root Cl^–^ content, **(D)** total Cl^–^ content, **(E)** leaf SO_4_^2–^ content, **(F)** shoot SO_4_^2–^ content, **(G)** root SO_4_^2–^ content, and **(H)** total SO_4_^2–^ content. The data are presented as means ± SD (*n* = 3). Significant differences between WT and *MdTyDc* overexpression lines are indicated by different lowercase letters based on Tukey’s multi-range test (*P* < 0.05). WT, wild type. OE-2, *MdTyDc* overexpression line 2. OE-3, *MdTyDc* overexpression line 3. OE-5, *MdTyDc* overexpression line 5.

Fifteen days of alkaline treatment reduced the leaf, stem, and root Cl^–^ content of WT plants (Figures 6A–D). However, under alkaline stress, leaf, root, and whole-plant Cl^–^ contents were significantly higher in OE lines than in WT (Figures 6A,C,D). Although the shoot Cl^–^ content of WT plants was higher than that of OE plants under normal and stress conditions, the Cl^–^ content of WT plants decreased significantly under stress conditions, whereas that of OE plants increased significantly (Figure 6B). The SO_4_^2–^ content of WT plants showed results similar to those for Cl^–^ content (Figures 6E–H). Under alkaline stress, there was no significant difference in leaf SO_4_^2–^ content between WT and OE plants (Figure 6E), and trends in shoot SO_4_^2–^ content of the genotypes were similar to those observed for Cl^–^ (Figure 6F). However, under alkaline conditions, the root and whole-plant SO_4_^2–^ contents of OE plants were considerably higher than those of WT plants (Figures 6G,H).

### *MdTyDc* Overexpression Improved N Metabolism Under Alkaline Stress

**FIGURE 7:**
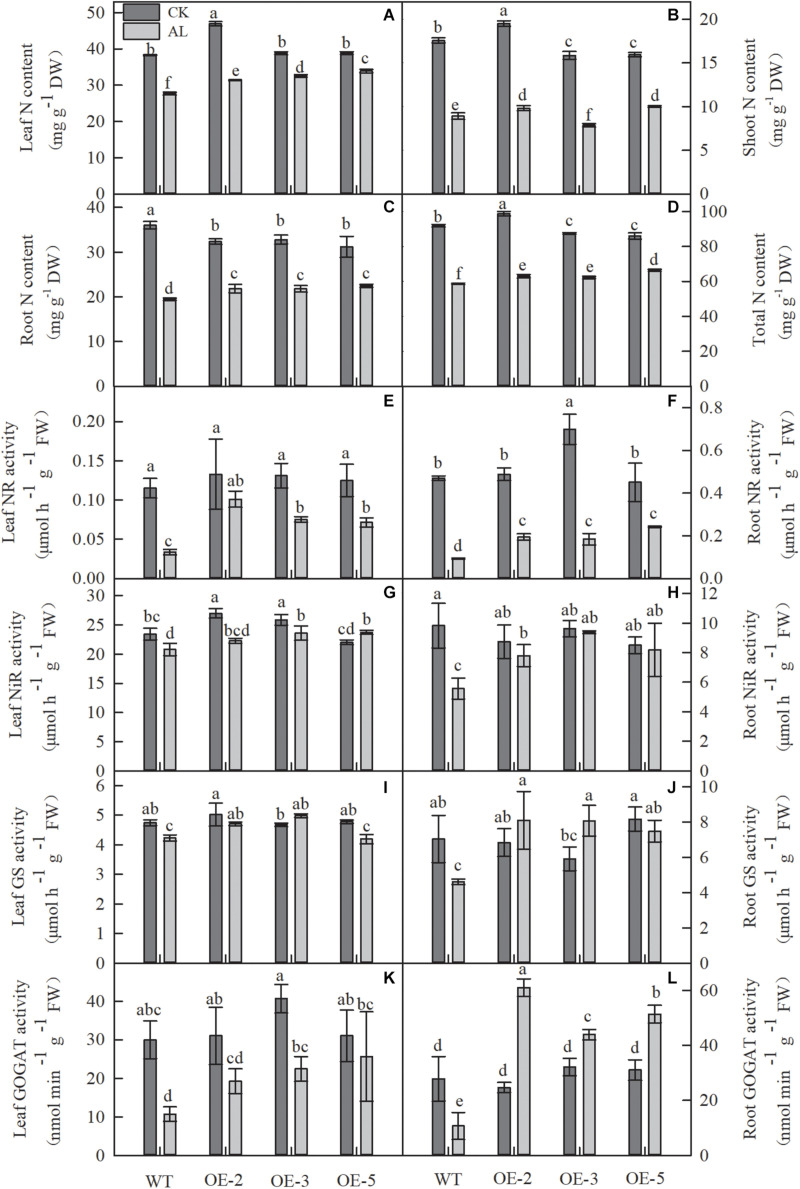
Alkaline stress caused less damage to the nitrogen content and the activity of enzymes involved in N metabolism in *MdTyDc* OE apples than in WT apples. **(A)** Leaf N content, **(B)** shoot N content, **(C)** root N content, and **(D)** total N content, **(E)** the activity of leaf NR, **(F)** root NR, **(G)** leaf NiR, **(H)** root NiR, **(I)** leaf GS, **(J)** root GS, **(K)** leaf GOGAT, and **(L)** root GOGAT. The data are presented as means ± SD (*n* = 3). Significant differences between WT and *MdTyDc* overexpression lines are indicated by different lowercase letters based on Tukey’s multi-range test (*P* < 0.05). WT, wild type. OE-2, *MdTyDc* overexpression line 2. OE-3, *MdTyDc* overexpression line 3. OE-5, *MdTyDc* overexpression line 5.

Because N metabolism is one of the most important metabolic activities in plant life, we measured the N content and the activities of enzymes related to N metabolism. Compared with normal growth conditions, growth under alkaline stress considerably reduced the N content in all apple plants (Figures 7A–D). However, overexpression of *MdTyDc* markedly enhanced leaf, root, and whole-plant N content of OE lines under stress conditions (Figures 7A,C,D). It is worth noting that the shoot N contents of OE-2 and OE-5 lines were 10.27 and 12.32% higher than that of WT under alkaline stress, but the shoot N content of OE-3 was 11.79% lower (Figure 7B).

After 15 days of alkaline treatment, the activities of NR, NiR, GS, and GOGAT were markedly lower than those measured under normal hydroponic conditions. Nevertheless, all these enzyme activities were considerably improved by the overexpression of *MdTyDc* under alkaline stress (Figures 7E–L). Interestingly, root GOGAT activity of all three OE lines was dramatically increased under alkaline stress compared with normal conditions (Figure 7L). These data indicated that *MdTyDc* overexpression in apple improved N metabolism under alkaline treatment, promoting the resistance of apple to alkaline stress.

### Overexpression of *MdTyDc* Affected the Expression of Genes Involved in Ion Transport, N Absorption and C Metabolism

**FIGURE 8:**
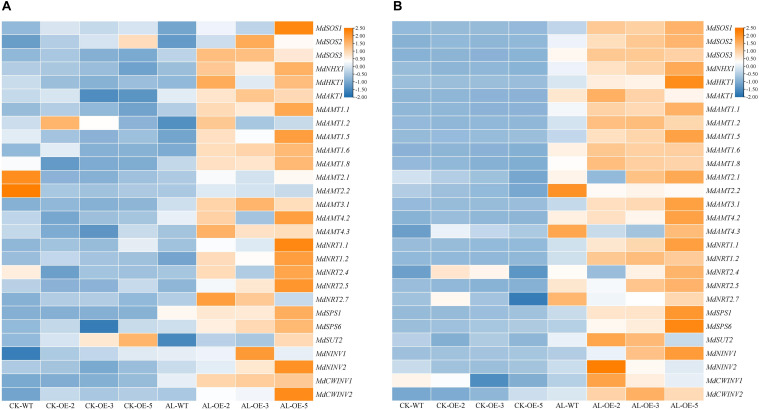
Heatmap presenting expression levels of 6 ion-transport-related genes (*MdSOS1*, *MdSOS2*, *MdSOS3*, *MdNHX1*, *MdHKT1*, and *MdAKT1*), 15 N-absorption-related genes (*MdAMT1.1*, *MdAMT1.2*, *MdAMT1.5*, *MdAMT1.6*, *MdAMT1.8*, *MdAMT2.1*, *MdAMT2.2*, *MdAMT3.1*, *MdAMT4.2*, *MdAMT4.3*, *MdNRT1.1*, *MdNRT1.2*, *MdNRT2.4*, *MdNRT2.5*, and *MdNRT2.7*) and 7 C-metabolism-related genes (*MdSPS1, MdSPS6*, *MdSUT2*, *MdNINV1, MdNINV2*, *MdCWINV1* and *MdCWINV2*) in **(A)** leaves and **(B)** roots of *MdTyDc*-overexpressing apple plants after 15 days under control and alkaline conditions. The data are presented as means ± SD (*n* = 3). Significant differences between WT and *MdTyDc* overexpression lines are indicated by different lowercase letters based on Tukey’s multi-range test (*P* < 0.05). WT, wild type. OE-2, *MdTyDc* overexpression line 2. OE-3, *MdTyDc* overexpression line 3. OE-5, *MdTyDc* overexpression line 5.

To further investigate the effect of *MdTyDc* overexpression on apple growth under alkaline stress, we measured the expression of genes related to ion transport and N absorption (Figure 8).

In leaves (Figure 8A), the expression of genes from the SOS signaling system (e.g., *MdSOS1*, *MdSOS2*, *MdSOS3*, and *MdNHX1*) was higher in at least two OE lines compared with WT plants under alkaline stress. The expression patterns of other ion-transport-related genes such as *MdHKT1* and *MdAKT1* were similar to those of *MdSOS3*. Moreover, with the exceptions of *MdAMT1.2*, *MdAMT2.1*, and *MdAMT2.2*, the transcript levels of other N-absorption-related genes (i.e., *MdAMT1.1*, *MdAMT1.5*, *MdAMT1.6*, *MdAMT1.8*, *MdAMT3.1*, *MdAMT4.2*, *MdAMT4.3*, *MdNRT1.1*, *MdNRT1.2*, *MdNRT2.4*, *MdNRT2.5*, and *MdNRT2.7*) were higher in OE lines than in WT lines after 15 days of alkaline stress. In addition, under alkaline stress, the expression of genes involed in C metabolism (e.g., *MdSPS1*, *MdSPS6*, *MdNINV2*, and *MdCWINV1*) was higher in OE plants than that in WT plants.

In roots (Figure 8B), the transcript levels of all ion-transport-related genes and N-absorption-related genes were up-regulated in WT plants under alkaline stress compared with normal growth conditions. With the exceptions of *MdAMT2.2*, *MdAMT4.2*, *MdAMT4.3*, *MdNRT2.4*, and *MdNRT2.7*, whose expression was lower in at least two OE lines than in WT lines after alkaline treatment, the other measured genes were all significantly upregulated in at least two OE lines. Furthermore, the *MdSPS1*, *MdSPS6*, *MdSUT2*, *MdNINV1*, *MdNINV2*, *MdCWINV1*, and *MdCWINV2* genes in at least two OE plants were more strongly induced by alkaline stress than those in WT.

## Discussion

Tyrosine decarboxylase (EC 4.1.1.25) is a 5’-pyridoxal phosphate (PLP)-dependent decarboxylase. Many studies have shown that it plays an active role in plant growth and development (Kang et al., 2007), secondary metabolite synthesis (Lan et al., 2013), and resistance to biotic and abiotic stresses (Landtag et al., 2002; Wang et al., 2020). Tyrosine is decarboxylated to tyramine by TYDC and then hydroxylated to dopamine, which is involved in plant response to salt stress (Li et al., 2015; Gao et al., 2020a), drought stress (Gao et al., 2020b), and low N stress (Liu et al., 2020). However, little is known about the function of *MdTyDc* in response to alkaline stress in plants. We found that *MdTyDc* expression was induced by alkaline stress (Figure 1A) and analyzed its role in the alkaline resistance mechanisms of apple using a hydroponic alkaline treatment.

As found previously, the inhibitory effect of alkaline stress on apple seedlings was significantly alleviated by the application of exogenous dopamine (Jiao et al., 2019). Here, we found that *MdTyDc* overexpressing apple lines, which showed higher dopamine content under both normal and alkaline conditions, exhibited enhanced alkalinity tolerance (Figures 1C–E and Table 1). For example, compared with WT plants under normal conditions, WT and OE plants grown under alkaline stress showed notable reductions in all growth parameters (shoot height, leaf number, fresh weight, dry weight, and RGR) (Table 1). However, the overexpression of *MdTyDc* clearly mitigated these declines. The root-shoot ratio of plants tends to increase in adverse environments, representing plants’ self-adaptation to the environment (Xia et al., 2010). We observed that *MdTyDc* overexpressing plants grown under alkaline stress had RSRs closer to those observed under normal conditions (Table 1). These findings indicate that the overexpression of *MdTyDc* may increase the dopamine content, improving plant growth and reducing the damage caused by alkaline stress.

The root system is a vital plant part that directly senses adversity signals, and measuring root system architecture is an effective way to quantify root development (Zhao et al., 2016). Alkaline stress can cause root growth retardation and directly or indirectly inhibit aboveground growth (Hu et al., 2012; Fan et al., 2015). Our experiment confirmed previous research in which almost all root system indices were seriously affected by alkaline stress (Table 2). Nevertheless, overexpression of *MdTyDc* could alleviate this inhibitory effect (Table 2). We also found that root activity was higher in OE plants than in WT plants (Figure 2A); root activity also reflects root growth and directly affects plant stress tolerance (Liu et al., 2019b). We therefore concluded that the root systems of plants that overexpressed *MdTyDc* showed better development under alkaline stress.

Numerous studies have shown that plant photosynthetic ability decreases under alkaline stress and that this decrease is proportional to the degree of stress (Liu et al., 2010; Liu and Shi, 2010). Here, Pn, Gs, and Tr of all genotypes decreased significantly from the 5th day of alkaline stress treatment, but this decrease was not significant in the three OE lines (Figures 3B,F,H). Measurements of Ci showed the opposite trend (Figure 3D), and this result suggests that the photosynthetic decline was due mainly to non-stomatal limitation (Huo et al., 2020). Furthermore, the decrease in photosynthetic ability caused by alkaline stress directly affects the photosynthetic apparatus by reducing chlorophyll content and Fv/Fm (Li et al., 2020a). Chlorophyll fluorescence, which can be quantified by Fv/Fm, reflects the true photosynthetic behavior of whole plants under adversity (Baker, 2008; Huang et al., 2020). Successive measurements during alkaline treatment showed that OE lines had higher chlorophyll contents and Fv/Fm values than WT lines under stress conditions (Figure 4). Many studies have shown that dopamine has a significant role in the regulation of oxygen reduction and the promotion of energy conversion during photosynthesis (Kanazawa and Sakakibara, 2000). Dopamine can also mitigate salt-induced photosynthetic limitations directly by reducing non-stomatal limitations associated with photosynthetic metabolism or indirectly by alleviating oxidative stress (Li et al., 2015). Considering the dramatic changes in photosynthesis on the fifth day after alkaline stress treatment, we hypothesized that there might be a significant change in dopamine content at this time. Therefore, we measured dopamine content in leaves and roots of apple plants after 5 days of alkaline stress treatment. Consistent with the study of Wang et al. (2020), dopamine content increased when *MdTyDc* was overexpressed in apples (Figures 1D,E and Supplementary Figure S1). In addition, dopamine content in OE plants under alkaline stress was higher than that under normal conditions. We therefore speculated that the overexpression of *MdTyDc* promoted the synthesis of dopamine, thereby alleviating damage to the photosynthetic system caused by alkaline stress in apple.

Guo et al. (2015b) found that the effect of alkaline stress on plant membranes was mainly manifested by an increase in REL. Therefore, we measured leaf REL and discovered that the overexpression of *MdTyDc* contributed to lower REL and maintained appropriate cell membrane permeability in apple under alkaline stress (Figure 2B). Proline, a compatible osmolyte present in the cytoplasm, can protect macromolecules and scavenge free radicals (Gong et al., 2014). Our research showed that *MdTyDc* overexpression promoted the accumulation of proline under alkaline stress (Figures 2C,D). This is consistent with previous research in which improved plant osmotic regulation helped to prevent the damage associated with alkaline stress (Ashraf and Foolad, 2007).

Excess Na^+^ accumulation under alkaline stress can damage membrane structure and alter membrane function, thereby increasing plasma membrane permeability and causing the exosmosis of intracellular potassium, phosphorus, and organic solutes (Tuna et al., 2007). Numerous studies have shown that changes in Na^+^ and K^+^ concentrations are a physiological means by which plants respond to alkaline stress (Shuyskaya et al., 2014; Abbas and Mobin, 2016). Here, apple plants that overexpressed *MdTyDc* maintained lower leaf, stem, and root Na^+^/K^+^ ratios than WT plants under alkaline stress (Figure 5). Therefore, plants under alkaline stress can effectively control the absorption and transport of Na^+^ and K^+^ and maintain Na^+^/K^+^ balance, which is important for adaptation to alkaline stress (Guo et al., 2016; Gresh et al., 2017). It has been reported that a sudden increase in Na^+^ in plant tissues under alkaline stress may also be related to the interference with the SOS signaling system (Türkan and Demiral, 2009). We found that the expression of SOS signal system genes (*MdSOS1*, *MdSOS2*, *MdSOS3*, and *MdNHX1*) was upregulated by *MdTyDc* overexpression under alkaline conditions (Figure 8). We also analyzed the expression of genes encoding potassium ion transporters or channels (*MdHKT1* and *MdAKT1*) and found that overexpression of *MdTyDc* upregulated their expression under alkaline stress (Figure 8). Previously, Li et al. (2015) found that dopamine regulated the expression of *MdSOS1*, *MdSOS2*, *MdSOS3*, *MdNHX1*, and *MdHKT1* under salt stress, helping to maintain a high K^+^/Na^+^ ratio and alleviating the damage caused by salt stress. Because Na^+^–K^+^ equilibrium is thought to be the ultimate manifestation of plant resistance to alkalinity (Qi and Spalding, 2004; Song et al., 2015; Wang et al., 2015b; Zhang et al., 2015), we hypothesized that *MdTyDc* overexpression would help to maintain a normal Na^+^/K^+^ ratio in cells by regulating the expression of genes related to Na^+^ and K^+^ transport.

The maintenance of intracellular ion balance and pH stability are necessary to ensure the normal progress of various metabolic processes (Yang et al., 2007). Here, we observed that more Cl^–^ and SO_4_^2–^ accumulated in OE lines than in WT lines under alkaline stress (Figures 6A–H). As previous studies have shown, plants absorb inorganic anions such as Cl^–^ and SO_4_^2–^ to balance the accumulation of excess cations under alkaline stress, thereby maintaining a stable pH (Ghoulam et al., 2002; Yang et al., 2007). Our results suggest that *MdTyDc* plays a role in plant ion balance and osmotic regulation.

Previous studies have shown that an adequate N supply helps to compensate for plant nutrient imbalances caused by alkaline stress (Ehlting et al., 2007; Wang et al., 2012a). The growth rate of trees leveled out when soil nitrogen concentration was highest (Vestin et al., 2013). Yang et al. (2007) showed that NO_3_^–^ absorption by an herbaceous halophyte was reduced due to alkaline stress, and we demonstrated that *MdTyDc* overexpression significantly alleviated reduced N content in leaves and roots (Figures 7A–D). Decreased N content under alkaline stress may be due to inhibition of N absorption and ammonium assimilation (Guo et al., 2015b). We therefore examined the activities of N metabolism enzymes and found that *MdTyDc* overexpression noticeably increased the activities of NR, NiR, GS, and GOGAT in leaves and roots (Figures 7E–L). Plant N uptake depends on the activity of corresponding carrier proteins (Munns and Tester, 2008). We measured transcript levels of genes related to N absorption and found that almost all N metabolism genes were induced by alkaline treatment in the roots of all genotypes (Figure 8B). This is consistent with the findings of Wang et al. (2012b), in which the *OsAMT* and *OsNRT* gene families played an important role in nitrate accumulation in old and new rice leaves under alkaline stress. Moreover, the expression of those genes was higher in plants that overexpressed *MdTyDc* than in WT (Figure 8). Taken together, our results suggest that *MdTyDc* overexpression increases the activities of enzymes related to nitrogen metabolism by regulating genes associated with N absorption and assimilation, thereby improving plant N content under alkaline stress.

It is well known that plants can accumulate and synthesize compatible small molecules such as sugars, alcohols, quaternary amines, proline and betaine in response to osmotic stress during saline-alkali stress (Flowers and Colmer, 2008). Coincidentally, previous studies have shown that overexpression of dopamine synthesizing genes in potatoes increases soluble sugar content (Swiedrych et al., 2004). The overexpression of dopamine receptors in potatoes increases the content of catecholamines, along with the content of sucrose, glucose, and fructose (Skirycz et al., 2005). In plants, sucrose can be converted to fructose and glucose by sucrose phosphate synthase (SPS), cell wall invertase (CWINV), and neutral invertase (NINV) (Diana et al., 2008; Hirose et al., 2008). Therefore, we examined the expression levels of several carbon metabolism-related genes (*MdSPS1, MdSPS6, MdSUT2, MdNINV1, MdNINV2, MdCWINV1, and MdCWINV2*) in leaves and roots. We found that the expression of *MdSPS1*and *MdSPS6* in the leaves and roots of OE plants were more strongly induced by alkaline stress than those in WT (Figure 8). Therefore, we hypothesized that *MdTyDc* overexpression may increase glucose and fructose synthesis by increasing the expression of *MdSPS1*/*6* thus improving the osmotic regulation ability of plants to resist alkali stress.

**FIGURE 9:**
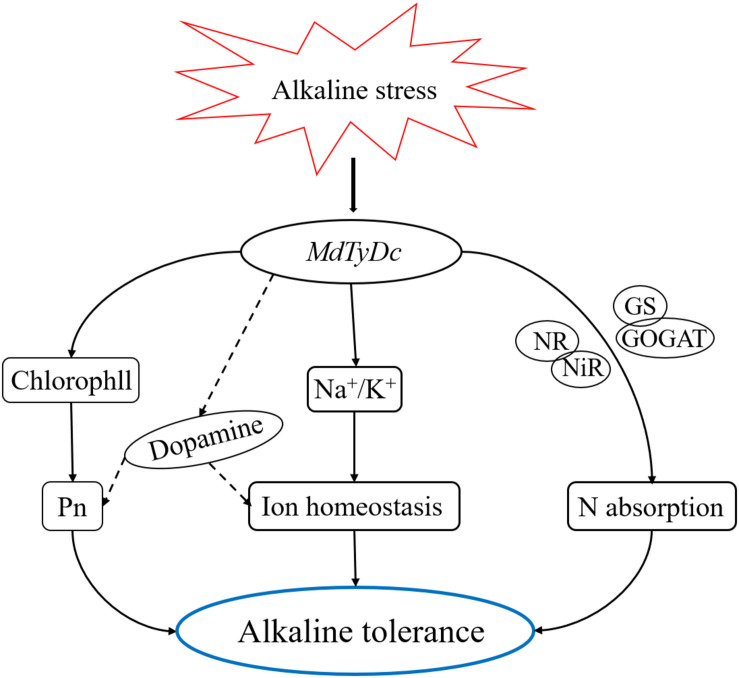
A model to explain the regulatory effect of *MdTyDc* on apple’s alkaline stress response. Under alkaline stress, apple lines overexpressing *MdTyDc* showed increased photosynthetic pigment content and inhibited the increase of sodium ions, thus the photosynthetic capacity was enhanced and intracellular ion homeostasis was maintained, which may be realized by increasing dopamine content; In addition, overexpression of *MdTyDc* also improved the enzyme activity related to nitrogen metabolism, thus promoting nitrogen absorption. Therefore, the alkaline tolerance of transgenic apple plants was increased.

In conclusion, our study demonstrated that apple plants overexpressing *MdTyDc* showed enhanced tolerance to alkaline stress. As shown in Figure 9, the exposure of *MdTyDc* overexpressing apple lines to alkaline stress led to higher dopamine content, which appeared to enhance photosynthetic capacity and maintain intracellular ion homeostasis. In addition, overexpression of *MdTyDc* promoted root development and increased the activities of enzymes related to N metabolism, which may have contributed to better plant N absorption under alkaline stress. Our study provides evidence for dopamine mediation of alkalinity tolerance and has important applications for promoting the growth of horticultural crops in saline and alkaline soils.

## Data Availability Statement

The original contributions presented in the study are included in the article/Supplementary Material, further inquiries can be directed to the corresponding author/s.

## Author Contributions

XL contributed to the study conception and performed most of the experiments. YJ and KT prepared the materials and obtained the experimental data. JZ, TG, ZZ, and YZ analyzed the data. FM contributed to the study design. CL provided all financial support, critical intellectual input in study design, and manuscript preparation. XL wrote the first draft of the manuscript. All authors commented on previous versions of the manuscript, read and approved the final manuscript.

## Conflict of Interest

The authors declare that the research was conducted in the absence of any commercial or financial relationships that could be construed as a potential conflict of interest.

## Supplementary Material

Supplementary Figure 1 Dopamine content of *MdTyDc*-overexpressing apple plants after 5 days under control and alkaline conditions. **(A)** Dopamine content in leaves and **(B)** dopamine content in roots. The data are presented as means ± SD (*n* = 3). Significant differences between WT and *MdTyDc* overexpression lines are indicated by different lowercase letters based on Tukey’s multi-range test (*P* < 0.05). WT, wild type. OE-2, *MdTyDc* overexpression line 2. OE-3, *MdTyDc* overexpression line 3. OE-5, *MdTyDc* overexpression line 5.
