# Supplementary figures and images for: MdTyDc Overexpression Improves Alkalinity Tolerance in Malus domestica
Source: Front Plant Sci. 2021 Feb 16;12:625890. doi: 10.3389/fpls.2021.625890 (PMC7921794; doi:10.3389/fpls.2021.625890)

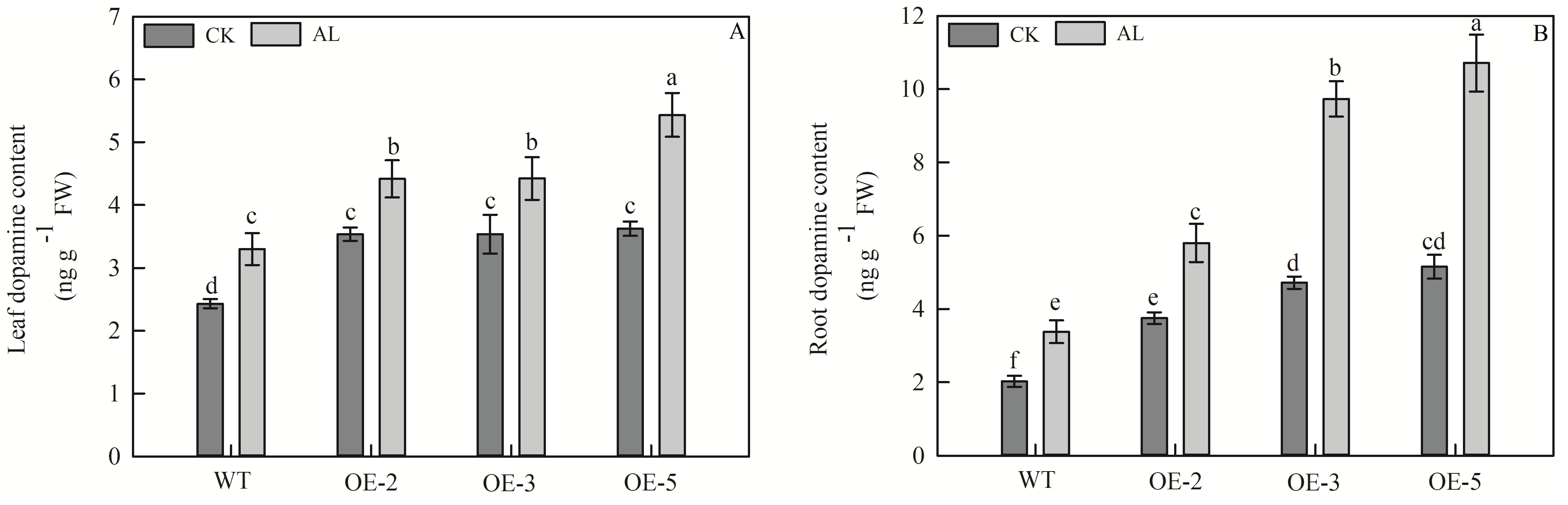

Supplement: Supplementary Figure 1 — Dopamine content of MdTyDc-overexpressing apple plants after 5 days under control and alkaline conditions. (A) Dopamine content in leaves and (B) dopamine content in roots. The data are presented as means ± SD (n = 3). Significant differences between WT and MdTyDc overexpression lines are indicated by different lowercase letters based on Tukey’s multi-range test (P < 0.05). WT, wild type. OE-2, MdTyDc overexpression line 2. OE-3, MdTyDc overexpression line 3. OE-5, MdTyDc overexpression line 5. [file Image_1.tif]
