# Supplementary material for: MdTyDc Overexpression Improves Alkalinity Tolerance in Malus domestica
Source: Front Plant Sci. 2021 Feb 16;12:625890. doi: 10.3389/fpls.2021.625890 (PMC7921794; doi:10.3389/fpls.2021.625890)
Supplement: Supplementary file 2 [file Table_1.docx]

***MdTyDc* overexpression improves alkalinity tolerance in *Malus domestica***

**Supplementary materials**

**TABLE S1 | Sequences of primers used in qRT-PCR**

| Gene | Primer sequence (5’-3’) |
| --- | --- |
| *MdTyDc*-F | AACAGCAGAAGATGAGTGGGT |
| *MdTyDc*-R | CATATCCTAAAAACTAAAGAGGTGC |
| *MdSOS1*-F | TCCGGTTAATCCATCACACACCGT |
| *MdSOS1*-R | TTTGCTGCCCTGGAGGATTTGTTG |
| *MdSOS2*-F | ACACGGGGAGGTAGTGACAA |
| *MdSOS2*-R | CCTCCAATGGATCCTCGTTA |
| *MdSOS3*-F | ATGTTGGTTTGTTAATGGGCTGC |
| *MdSOS3*-R | CGTTCACAGTAAAAGGTGTCGCA |
| *MdNHX1*-F | AAGCGACAGTCCTGGAACATCAGT |
| *MdNHX1*-R | TATTATCACTTGCTGCCGGAGGCT |
| *MdHKT1*-F | TCGTTCGCTATTTCGTGTCCTGCT |
| *MdHKT1*-R | TGGGCCTGAAAGAAGTGTTTGTGC |
| *MdAKT1*-F | ATTCAAGTATGCCTGGGTGC |
| *MdAKT1*-R | TTGTAAACCTCCCAAGGAACCGGA |
| *MdAMT1.1*-F | ATCCGACGCCAACTACACTTCTAC |
| *MdAMT1.1*-R | CGATTTGGTCACAGATGAAGTGAG |

| *MdAMT1.2*-F | AAGCGAGGATGAGACTCAAGGG |
| --- | --- |
| *MdAMT1.2*-R | GCAGGACTGGCATCATTAACAGG |
| *MdAMT1.5*-F | ACAGATAGTGGTAATTATAGGGTGGG |
| *MdAMT1.5*-R | CGTGGTCATGGTACACGTAAGC |
| *MdAMT1.6*-F | GTAACTTATTGTTCACCTCTGGGGC |
| *MdAMT1.6*-R | CGTTCCTGAACGCGTCGAAT |
| *MdAMT1.8*-F | GTTGTTAGGCTCTGGAGCCATTG |
| *MdAMT1.8*-R | GCCTTCCCAAACGTGTCAAAC |
| *MdAMT2.1*-F | GTGACGATGGATCGATTGAGACTC |
| *MdAMT2.1*-R | CCCGCTAACAAAATAAGAGTAATAGCT |
| *MdAMT2.2*-F | ATATCGGAGATGACGCGGTTC |
| *MdAMT2.2*-R | CGTACGCTGCTGTATTCCGAC |
| *MdAMT3.1*-F | TCACTGGCCTCGTCTGCATTAC |
| *MdAMT3.1*-R | GGTGTCGTCAACTGCGGATAAC |
| *MdAMT4.2*-F | GGAGGTCCCAACGTGCACAC |
| *MdAMT4.2*-R | CACGATGAACCGAATCAGAAAAC |
| *MdAMT4.3*-F | ATCACTGGCTTAGTTTGCATTACTC |
| *MdAMT4.3*-R | GACTTGCTTTAGGATGCCGATT |
| *MdNRT1.1*-F | CTGGCTGGTCCCACAGTTCTT |
| *MdNRT1.1*-R | CTTCATTCCTTTCGGGCACTC |
| *MdNRT1.2*-F | TTAATTGCTGCCACACTTCATAG |
| *MdNRT1.2*-R | CACGATGTTTGGTTCTGATACTTC |
| *MdNRT2.4*-F | CAGAAGGTGAACCCGGAAG |

| *MdNRT2.4*-R | | CAAGTGGAACGTCCTCATGTG |  |  |
| --- | --- | --- | --- | --- |
| *MdNRT2.5*-F | | TTGTGGTCCATCTAAGAACAAGGC |  |  |
| *MdNRT2.5*-R | | TCATCAGAGGGTCGGGTAACAG |  |  |
| *MdNRT2.7*-F | | TCTCCAGGCAGACGAGCATT |  |  |
| *MdNRT2.7*-R | | GGAGCAAGTGATACTGGTTTGTTTC |  |  |
| *MdSPS1*-F | AGTGTAGTACTCAAGGGAGTTGG | |  |  |
| *MdSPS1*-R | TGCTCATGGGGAAGGCTTTAC | |  |  |
| *MdSPS6*-F | | | AGGTTCTGTTGAGTATGGCAGTGAG | |
| *MdSPS6*-R | | | GTGCTTCAAGTGCCGCTGAGA | |
| *MdCWINV1*-F | | | TAACAAATATGTGGTGCTCCTCTG | |
| *MdCWINV1*-R | | | ACCCTAGCTGTTATGCACGCCT | |
| *MdCWINV2*-F | | | TTCAAAGCTAAAGGCAGACACG | |
| *MdCWINV2*-R | | | GTAAATCTACATCTACAAAGCCAGC | |
| *MdNINV1*-F | | | GTCCATTGTTTCATCATTGGGTAC | |
| *MdNINV1*-R | | | GGTCGCTGCCAGTGATTATACG | |
| *MdNINV2*-F | | | GAGTTCCAGACAGGCATAAGGCT | |
| *MdNINV2*-R | | | CCATCCGTCTATCAATCATACAGG | |
| *MdSUT2*-F | | | ACTCACTATGTATCAGCAGAAT | |
| *MdSUT2*-R | | | TGAGATGGCCTCCTTTAGATTCT | |
| *MdMDH*-F | | CGTGATTGGGTACTTGGAAC |  |  |
| *MdMDH*-R | | TGGCAAGTGACTGGGAATGA |  |  |
|  | |  |  |  |
|  | |  |  |  |
|  | |  |  |  |
|  | |  |  |  |
|  | |  |  |  |
|  | |  |  |  |
|  | |  |  |  |
|  | |  |  |  |
|  | |  |  |  |
|  | |  |  |  |
|  | |  |  |  |
|  | |  |  |  |
|  | |  |  |  |
